# Degradation of SARS-CoV-2 specific ribonucleic acid in samples for nucleic acid amplification detection

**DOI:** 10.1371/journal.pone.0264541

**Published:** 2022-03-11

**Authors:** Katsuyuki Takeuchi, Hiroyuki Yanagisawa, Yukiko Kurosawa, Yoritsugu Iida, Kosuke Kawai, Shigehiko Fujimaki

**Affiliations:** 1 Institute of Education, Innovative Human Resource Development Division, Tokyo Medical and Dental University, Bunkyo‐ku, Tokyo, Japan; 2 Department of Genetics, Hyogo College of Medicine, Nishinomiya, Hyogo, Japan; 3 Central Chemical Laboratory, SGS Japan Inc., Hodogaya-ku, Yokohama, Japan; New England Biolabs Inc, UNITED STATES

## Abstract

The degradation of SARS-CoV-2 specific ribonucleic acid (RNA) was investigated by a numerical modeling approach based on nucleic acid amplification test (NAAT) results utilizing the SmartAmp technique. The precision of the measurement was verified by the relative standard deviation (RSD) of repeated measurements at each calibration point. The precision and detection limits were found to be 6% RSD (seven repeated measurements) and 94 copies/tube, respectively, at the lowest calibration point. RNA degradation curves obtained from NAAT data on four different temperatures were in good agreement with the first-order reaction model. By referring to rate constants derived from the results, the Arrhenius model was applied to predict RNA degradation behavior. If the initial RNA concentration was high enough, such as in samples taken from infected bodies, the NAAT results were expected to be positive during testing. On the other hand, if initial RNA concentrations were relatively low, such as RNA in residual viruses on environmental surfaces, special attention should be paid to avoid false-negative results. The results obtained in this study provide a practical guide for RNA sample management in the NAAT of non-human samples.

## Introduction

The viral infection coronavirus disease 2019 (COVID-19) appeared in late 2019 and by 2020 had been classified a pandemic. The COVID-19 pandemic claimed millions of lives and infected hundreds of millions of people worldwide [1]. Recently, mass vaccination has mitigated COVID-19, yet SARS-CoV-2 variants have been discovered in many parts of the world [2–4]. Unfortunately, the pandemic is still dominating the globe, posing a threat to the global economy by causing lockdowns in cities, which causes blockages in multilateral logistics [5]. No one knows when humanity will overcome COVID-19. The nucleic acid amplification test (NAAT) is an effective method for identifying COVID-infected individuals and enables positive patients to be transported to isolation or medical facilities for proper treatment [6, 7].

The NAAT provides qualitative positive/negative results for detecting COVID-19 infection. False-negative results should be ruled out. However, ribonucleic acid (RNA) degradation is an unavoidable risk that is activated under wet conditions depending on ambient temperatures [8]. A possible error is that RNA degradation in the inactivation solution after sampling provides false-negative result. If the initial RNA concentration is high enough, such as in samples obtained from infected bodies, NAAT results are expected to remain positive until the test concludes. On the other hand, when testing samples with relatively low RNA concentrations, the impact of RNA degradation on NAAT test results remains unclear and caution should be taken to exclude false-negative results. In the scenario of concern for RNA degradation, NAAT for residual viruses on environmental surfaces has become our main focus.

Many articles report that NAAT for the SARS-CoV-2 virus is the most effective preventative measure to combat the COVID-19 pandemic [9–11]. There have been a few studies of samples obtained from indoor materials to determine how to prevent viral contact infections [12]. However, those previous studies did not elucidate the details of RNA degradation behaviors. With this in mind, RNA degradation was investigated by a numerical modeling approach based on NAAT results. The purpose of this study was to quantitatively evaluate RNA degradation in samples for nucleic acid detection. This study was conducted with the aim to provide a practical guide for RNA sample management backed by quantitative analysis of RNA degradation behavior based on numerical models.

## Materials and methods

### RNA measurement

The golden standard for diagnosing COVID-19 is reverse transcription-polymerase chain reaction (RT-PCR) [13]. In general, RT-PCR is expensive, requires a trained laboratory technician, and takes time—3 to 4 hours per sample—compared to rapid antigen testing. However, rapid antigen testing is less reliable than NAAT [14]. Therefore, rapid antigen testing has become uncommon as the demand for accurate COVID-19 testing increases. In recent years, other NAAT techniques, e.g. isothermal nucleic acid detection methods such as SmartAmp [15] or loop mediated isothermal amplification (LAMP) [16, 17] have been adopted for the operability and miniaturization of equipment. In this study, experimental data on the degradation of SARS-CoV-2 specific RNA in various samples were collected using a portable NAAT device with a unique isothermal amplification technique (SmartAmp method) suitable for on-site testing. Details of the experimental method, materials, and reagents are as follows. Artificially synthesized RNA was used as an alternative to actual RNA in the SARS-COV-2 RNA test due to safety concerns.

### Reagents

A SmartAmp SARS-CoV-2 kit commercialized by DANAFORM Inc. (Japan) in July 2021 for diagnosing COVID-19 was used. The effectiveness and validity of the SmartAmp assay for the diagnosis of COVID-19 was validated by Asai et al. [18]. SmartAmp Reagents Kits (RR005, polymerase, nucleoside triphosphate, fluorescent probe, and standard RNA solution) and a Smart Extract (RR004, inactivation solution, washing solution, column, and collection tube) were purchased from DNAFORM Inc. (Japan) for SmartAmp. Our sequence of the standard RNA containing 400 ribonucleotides (adjusted to 10,000 copies/μl) is detailed in supporting information (S1 Fig in S1 File). Other reagents, such as RNA-free water (diethylpyrocarbonate-treated water) and ethanol (99.5%), were purchased from Thermo Fisher Scientific (USA) and Kanto Chemical Co. Inc. (Japan), respectively. A 15 ml centrifuge tube (VIO-15BN), 0.2 ml eight-tube flat type, and 1.5 mL tube were purchased from AS ONE Corporation (Japan).

#### Instruments

A device package released by DNAFORM Inc. (Japan) as Life Case, including an RNA extractor, a real-time isothermal nucleic acid amplifier, a mini centrifuge (model Mini-6ks), and a circulator (model MiniT-C), were prepared. The real-time isothermal nucleic acid amplifier enables a test to be completed in 40 minutes with almost the same precision as a conventional PCR device. The Life Case package is intended for on-site testing, and all equipment and reagents required for the testing fit in three carry cases. In addition, a refrigerator (Medicool) for 4°C and a freezer (VT-78HC) for −80°C settings were purchased from SANYO Electric Co., Ltd. and Nihon Freezer Co., Ltd. (Japan), respectively.

#### RNA quantification

**Fig 1:**
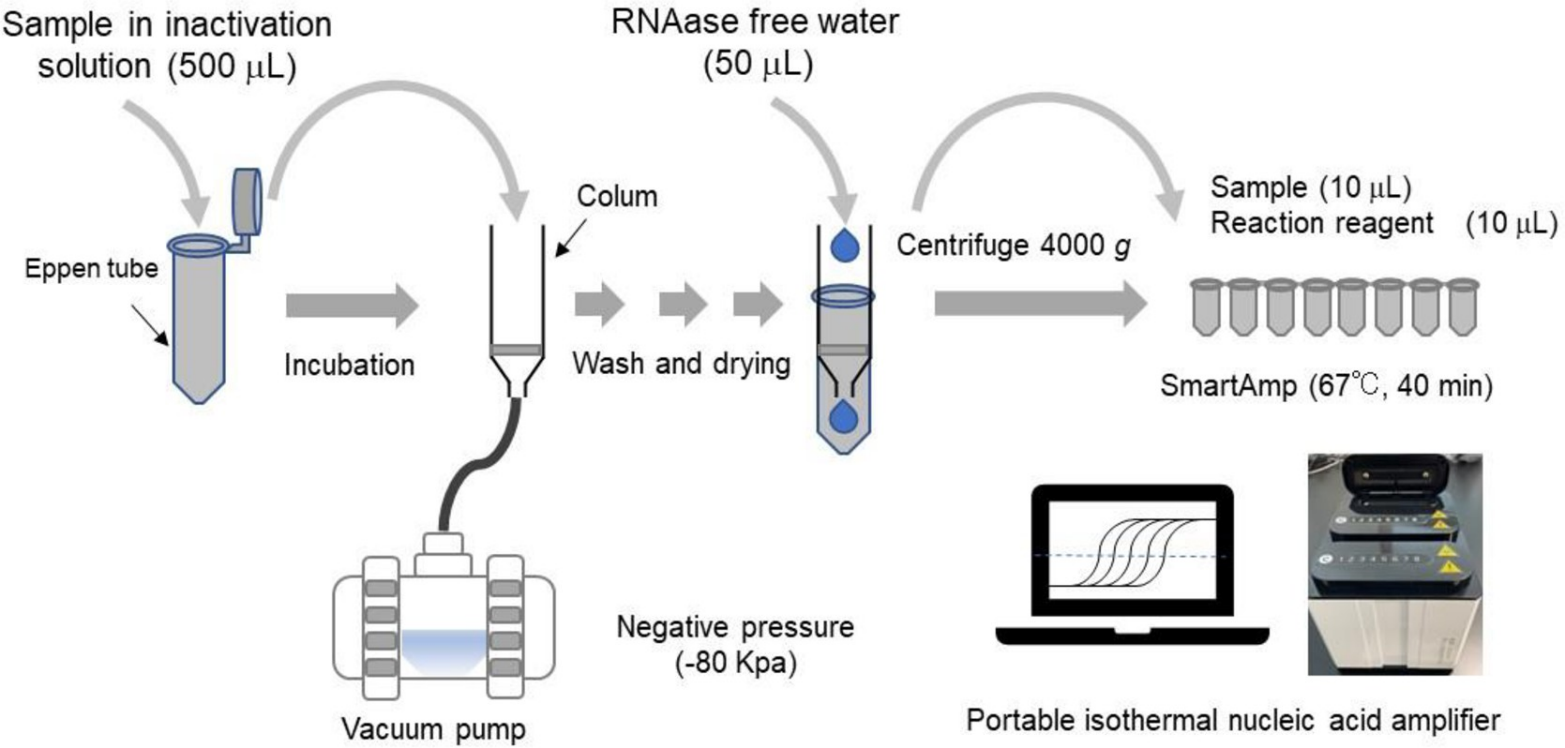
Schematic diagram of the RNA quantification procedure.

Swab samples are always treated with an inactivating agent for safety. Primarily, standard reagents were diluted on ice with an inactivation solution to 40 copies/ μl. Then, RNA samples were incubated using a circulator or refrigerator. RNA was purified using a smart extract kit for SmartAmp. Fig 1 illustrates the schematic diagram of the RNA quantification procedure. Secondly, 500 μl of the reagent (20,000 copies) was added to the glass filter column and aspirated the solution by a pump. Similarly, 1 ml of the washing solution was added to the column and aspirated, and then 1 ml of ethanol was added and aspirated for 3 minutes until the column was completely dried. Tertiary, 50 μl RNA-free water was added to the column and centrifuged at 4,000 rpm to extract the mixture. Quaternary, the RNA extract sample (10 μl, 4,000 copies) was amplified using Life Case under isothermal conditions of 67°C. Finally, the fluorescence intensity was monitored by a photon counter that enables detection of wavelengths 510–530 nm. The time at which the fluorescence signal crossed the threshold line set to 1/10 of the maximum value was used as a reference index C_t_.

The concentration of SARS-CoV-2–specific RNA was obtained from fluorescent signals calibrations. Fluorescent signals from the amplified nucleic acid were processed with a fluorescent photon counting profile and the concentration of SARS-CoV-2 specific RNA was determined in each sample. The RNA concentration was obtained by calibrating the C_t_ index determined from the SmartAmp assay profile.

### Calibration curve

To confirm the measurable range and repeatability of RNA in this experiment, 250, 500, 2,000, 5,000, and 10,000 copies/μl were prepared by diluting a standard RNA solution (10,000 copies/μl) with an inactivation solution on ice. The calibration was performed using the same standard solution as described above in “RNA quantification” to ensure reliable quantification results. Each of the 500 μl of the above solution is concentrated and finally extracted with water at 50 μl. Since 10 μl is used for single SmartAmp test, the number of copies will be 500, 1,000, 4,000, 10,000, 20,000 copies/tube, respectively.

### Numerical modeling

In this study, the first-order reaction model was chosen as the initial speculation of the RNA degradation pathway [19]. The RNA degradation rate *v* was assumed to proceed in a first-order reaction proportional to the reaction rate constant *k* with respect to the RNA concentration.

v=k∙[RNA] (1)

where v represents the degradation rate of $v=-\frac{d[RNA]}{dt}$

As shown below, numerical analysis of the RNA degradation reaction was performed using the integral form of this equation.

According to Eq (1), it can be shown as

$$\frac{d[RNA]}{\left[RNA\right]}=-k∙dt$$ (2)

Integration of Eq (2) yields

$$\frac{ln[RNA]}{{\left[RNA\right]}_{0}}=-k∙t,$$ (3)

which can be represented by the following formula.


$$\left[RNA]={\left[RNA\right]}_{0}∙exp(-k∙t\right)$$ (4)

In determining the reaction rate constant *k* from the experimental values, the nonlinear least-squares method was applied to Eq (4). The conjugate gradient method was used for the fitting algorithm.

Furthermore, the relationship between the reaction rate constant *k* and the absolute temperature *T* was derived using the Arrhenius model [20]. Either exponential nonlinear fitting or log-linear fitting was applied to the following equations, respectively.

$$k(T)=A∙exp(-\frac{B}{T})$$ (5)

$$\ln\;k(T)=lnA-B∙(\frac{1}{T})$$ (6)

where *A* and *B* are constants. *A* is the pre-exponential factor or frequency factor, and *B* is the constant associated with activation energy. The activation energy of the degradation reaction can be calculated by multiplying *B* by the gas constant.

## Results and discussion

### Calibration curve and repeatability in the SmartAmp method

**Fig 2:**
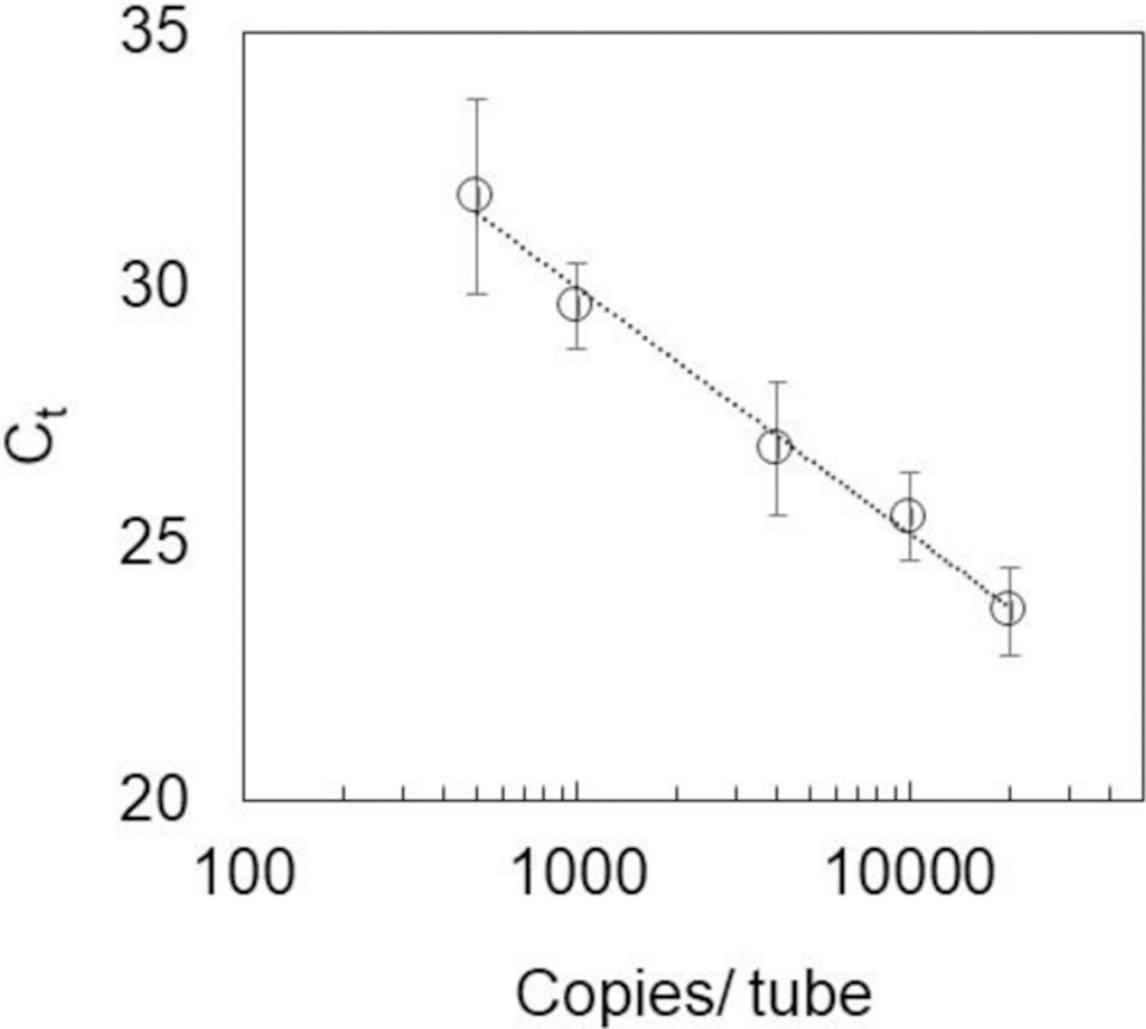
Calibration curve for RNA (preparation amount: 500, 1,000, 4,000, 10,000, 20,000 copies/tube).

Fig 2 shows a diagram in which the horizontal axis is the number of copies per tube in logarithmic, and the vertical axis is the *C*_*t*_. Results were obtained in which the *C*_*t*_ value was proportional to the amount of RNA copies/tube. Table 1 summarizes the experimental parameters, such as detection limit, RSD, and the coefficient of determination. The detection limit was calculated by multiplying the standard deviation obtained from seven independent experiments at 500 copies/tube by Student’s *t*-factor (3.14 for 99% confidence). The data show a sufficiently low detection limit (approximately 100 copies/tube), a high correlation coefficient (0.990), and a satisfactory RSD at 500 copies/tube (6.0%). The quantifiable range of this series of operations is in the range of 100 to 20,000 copies/tube. For the degradation efficiency experiment in the next section, 4,000 copies/tube was used in this study.

**Table 1:** Experimental parameters in RNA analysis.

Detection limit	RSD at 500 copies/tube	Coefficient of determination
94 copies/tube	6.0%	0.990

### Determining the rate constant of RNA degradation

**Fig 3:**
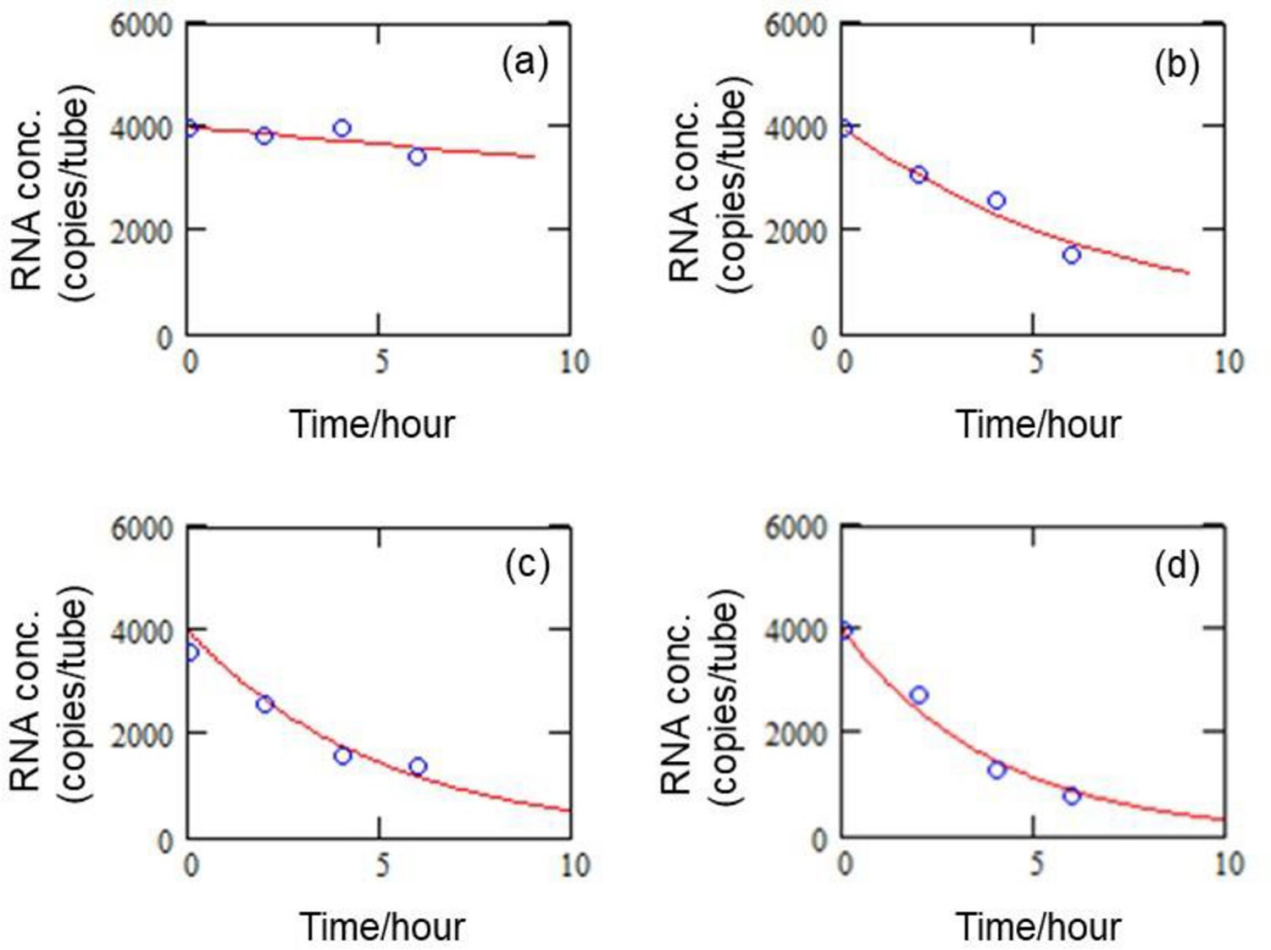
Time-dependent degradation of RNA at (a) 4°C (b) 25°C, (c) 37°C, and (d) 45°C.

Fig 3 shows the results of SmartAmp measurements in terms of time-dependent comparative degradation of RNA at four different temperatures of (a) 4°C, (b) 25°C, (c) 37°C, and (d) 45°C. The results given in Fig 3 as (a)–(d) are independent experiment replicates. The elapsed time (incubation time) started from the moment the sample was placed in the inactivated solution for SmartAmp testing.

As Fig 3 shows, the correlation curves fit well into the first-order reaction model. Table 2 shows the calculated rate constants obtained from nonlinear fitting to the Eq (4) by the least-square method, along with the half-life and time constant.

**Table 2:** Rate constants of RNA degradation reactions at various temperatures.

Temperature (°C)	Rate constant *k* (hr^–1^)	Half-life (hr)	Time constant (hr)
4	0.017	40.1	57.9
25	0.137	5.08	7.33
37	0.206	3.37	4.86
45	0.255	2.72	3.93

The rate constants shown in Table 2 quantitatively indicate that RNA degradation activity increases with temperature. These results demonstrate that a temperature-dependent reaction predominates the degradation pathway, at least between 4°C, and 45°C.

Half-life ($\tau =\frac{ln2}{k}$) is an indicator of the first-order reaction representing the time required for the concentration to decrease to half of the initial value, whereas, the time constant ($\tau =\frac{1}{k}$) is another index of the first-order reaction representing the time required for the concentration to decrease to $\frac{1}{e}$ of the initial value. Both indicators can be used as a guide for more practical management of RNA degradation. For example, when half-life is employed as the indicator, the RNA concentration reaches less than 0.1% of the initial value over a period of ten times the half-life (i.e., 50.8 hours at 25°C and 33.7 hours at 37°C).

A log-linear fitting was performed using the following equation to obtain the relationship between the reaction rate constant k and the temperature T.

$$\ln\;k(T)=lnA-B∙(\frac{1}{T})$$ (7)

where *A* and *B* are constants. *A* is the pre-exponential factor or frequency factor, and *B* is the constant associated with activation energy.

**Fig 4:**
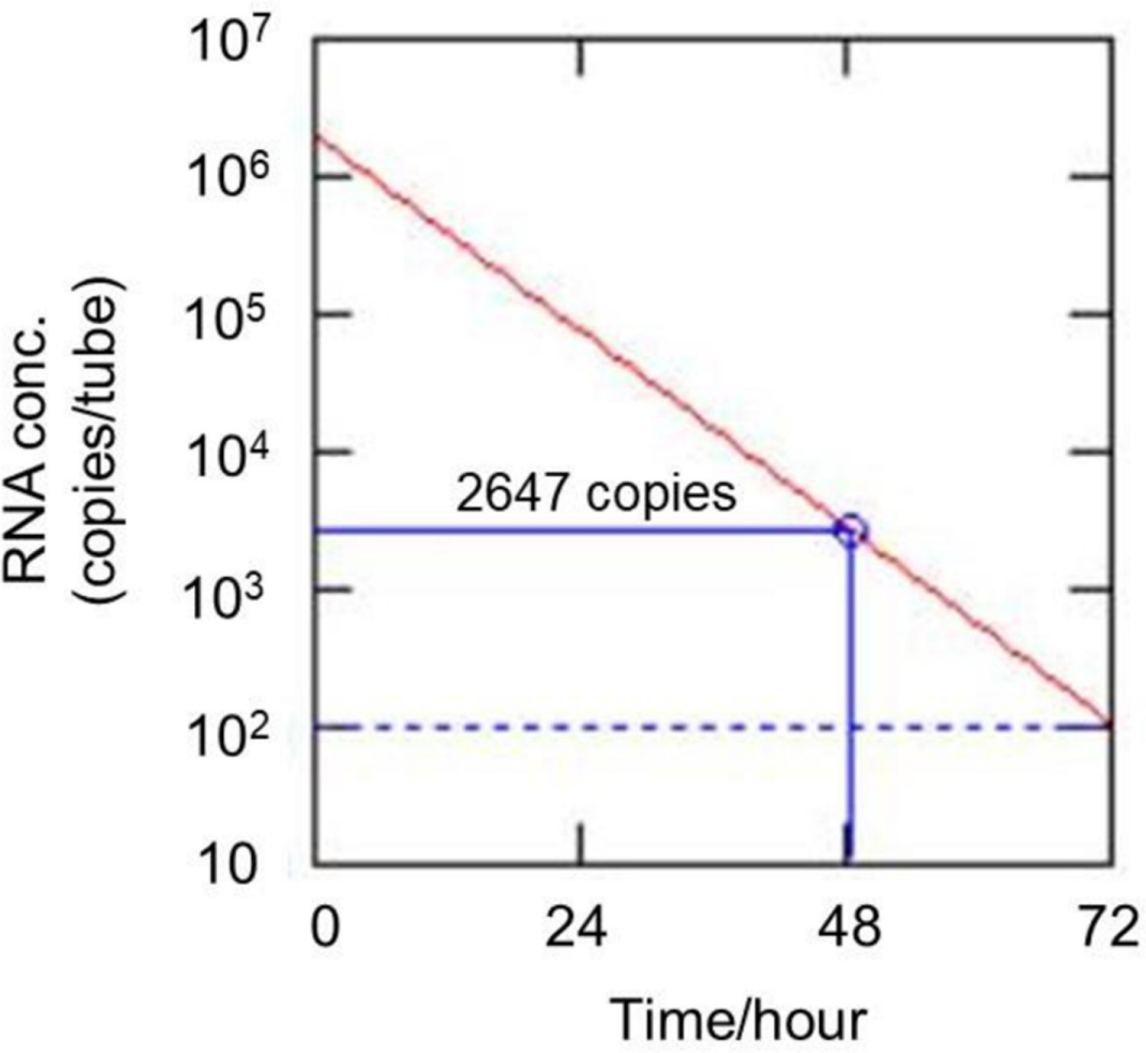
Over-time estimation in which RNA degrades to the detection limit at 25°C. The dotted line represents the detection limit.

Fig 4 shows an over-time estimation in which RNA degrades to the detection limit. As can be seen in Fig 4, RNA degrades from 2,647 copies to the detection limit (100 copies) in the last 24 hours, i.e. samples containing RNA less than 3,000 copies can end up with false-negative in 24 hours at 25°C. This is not limited to the environmental coronavirus, but also applicable to human coronavirus. However, for human coronavirus, samples are usually not left at room temperature for hours using storage refrigerators and shipping ice packs. This implies that special attention should be paid to samples with lower RNA levels if not tested for many hours at room temperature. RNA degradation is unavoidable, and the risk of false-negative results undoubtedly increases overtime at higher temperatures. In this regard, RNA remaining on environmental surfaces should be tested on-site immediately to minimize degradation, preferably using a portable device, e.g., Life Case kit. As mentioned earlier, the use of a portable type SmartAmp should shorten the measurement time and rule out false-negative results.

### Temperature-dependent RNA degradation

**Fig 5:**
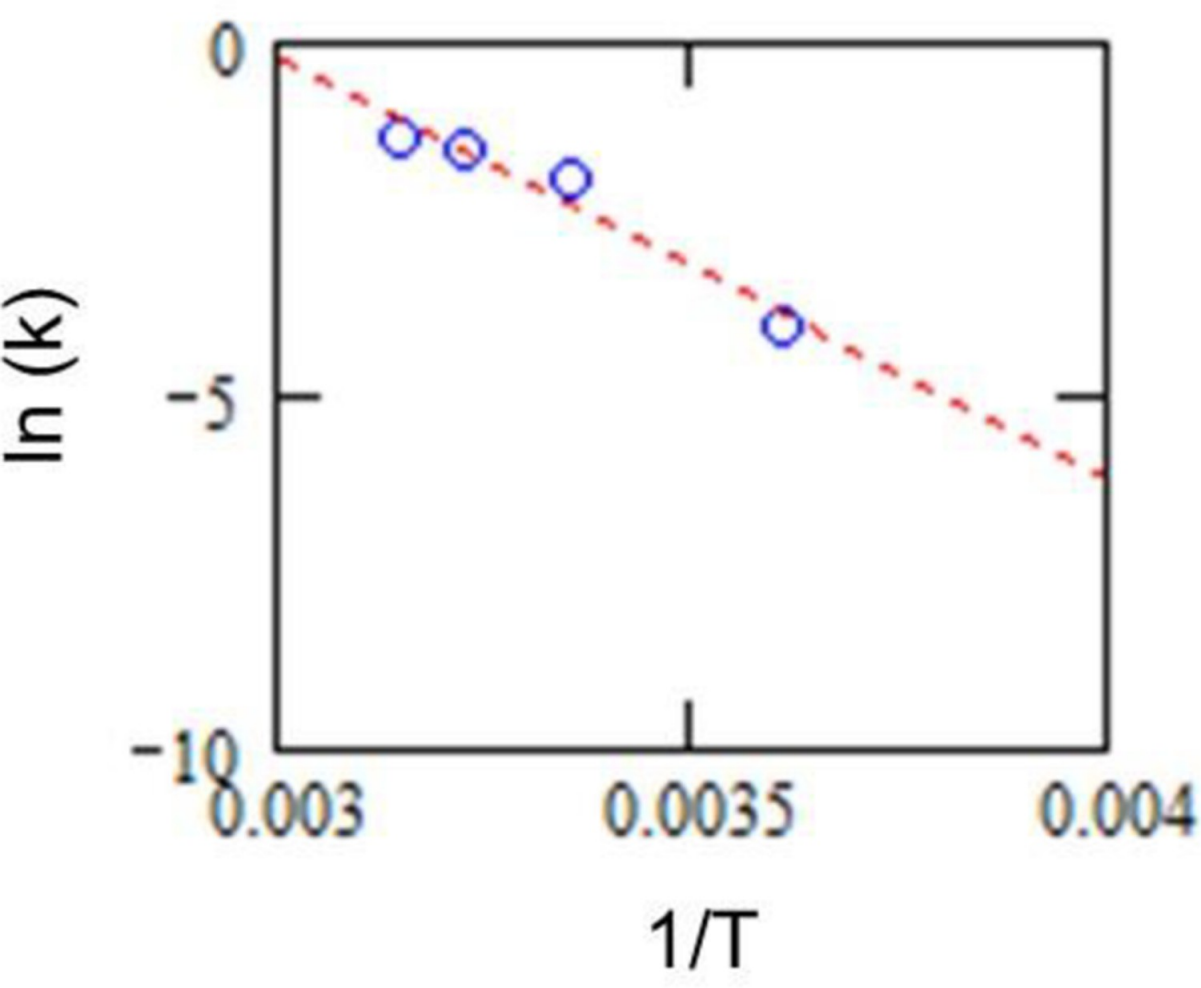
Relationship between the logarithm of reaction rate constant *k* and the inverse of temperatures *T*.

Based on the results in Table 2, the Arrhenius equation can represent the dependence of temperature on the reaction rate constant. The “log-linear fitting” in Fig 5 shows the relationship between the reaction rate constant *k* and the temperature *T*. The data plots fit well with the Arrhenius model.

These results enable us to estimate the reaction rate constant at any temperature and help predict RNA degradation over time. For NAAT tests on infectious viruses (e.g., SARS-CoV-2 virus), it is common for a qualitative positive/negative determination to be provided. Therefore, false-negative results should be ruled out. To rule out a false-negative result, rigid control of temperature while testing is required. Yet, degradation of RNA in an aqueous solution is subject to ambient temperature [7], and full temperature control cannot be achieved in the real world. RNA concentrations on environmental surfaces surrounding hospitalized patients were reported to range from 5.91 x 10^1^/swab to 2.19 x 10^5^/swab [21]. Another report found a median of 9.2 x 10^2^ copies/mL RNA in swabs sampled from patient equipment surfaces, and 3.3x 10^6^ and 9.2 x 10^7^ copies/mL RNA in swabs taken from the salivary pharynx of symptomatic patients [22]. With reference to these literatures, the copy number of viral RNA in environmental coronavirus should be lower than in human coronavirus (e.g., by more than two orders of magnitude), and the RNA copy count of environmental coronaviruses can be assumed to be somewhere below 10^6^ copies.

If the initial RNA concentration is high enough, e.g., in samples taken from infected bodies, the results remain positive while testing. Therefore, when testing residual viruses on environmental surfaces at relatively low concentrations, the results need to take RNA degradation into account.

The details of the RNA degradation mechanism remain unclear, however the activation energy of the major rate-determining reaction was estimated to approximately 50 kJ/mol from the Arrhenius model calculation. Arrhenius modeling indicates that higher temperature enhances the RNA degradation rate. Given such rapid RNA degradation under ambient temperature, our result provides a practical guide to RNA sample management in NAAT for residual viruses sampled from environmental surfaces under temperature-changing conditions.

### Sample and sampling conditions

In this study, artificially synthesized RNA was used as an alternative to actual RNA in the SARS-COV-2 RNA test for safety concerns. For real samples, the situation would be a little more complicated due to other factors that affect RNA degradation. Unfortunately, the initial RNA copy count for environmental coronaviruses can vary depending on sampling conditions. The possible factors that may affect the sampling conditions include the presence of RNA-degrading enzymes [23], hydrolysis on wet surfaces [24], and reagents remaining after antivirus treatment such as sodium hypochlorite [25]. Moreover, the status of COVID-19 patients in the vicinity of the sampling area, cleaning and disinfection, sampling procedures, detection methods, contamination rates, sample surface materials and sampling methods are also considered to be among the factors affecting the initial RNA concentration [26]. The negative effect of sampling conditions on NAAT results has not been incorporated into this study and can be considered the limitation of this paper. Further studies on sampling conditions remain as our future task.

## Conclusion

Degradation of SARS-CoV-2 specific RNA was investigated by a numerical modeling approach based on SmartAmp testing results. The precision of SmartAmp measurements was verified by the RSD of repeated measurements at each calibration point. The precision and detection limits were found to be 6% RSD (7 repeats) and 94 copies/tube (*t*-test 99% confidence), respectively, at the lowest calibration point. The RNA degradation curves obtained from the SmartAmp measurements on four different temperatures (4°C, 25°C, 37°C, and 45°C) were in good agreement with the first-order reaction model. The Arrhenius model was used to predict RNA degradation behavior by referring to reaction rate constants derived from the results. If the initial RNA concentration was high enough, such as samples taken from infected bodies, the NAAT results were expected to remain positive when testing. On the other hand, RNA concentration in residual viruses on environmental surfaces would be relatively low, special attention should be paid to avoid false-negative results. This study provides a practical guide to RNA sample management in NAAT of non-human samples. The activation energy of the major rate-determining reaction was estimated to be approximately 50 kJ/ mol. Pursuit of RNA degradation mechanisms is ongoing and has been left to future research. The present article does not incorporate the negative effects of sampling conditions on NAAT results and can be considered the limitation of this paper. Further studies on sampling conditions remain as our future task.

## Supporting information

S1 File Standard RNA sequence: NSP15 (400 bp). (DOCX)
