# Supplementary material for: Degradation of SARS-CoV-2 specific ribonucleic acid in samples for nucleic acid amplification detection
Source: PLoS One. 2022 Mar 11;17(3):e0264541. doi: 10.1371/journal.pone.0264541 (PMC8916628; doi:10.1371/journal.pone.0264541)

S1 File

**Degradation of SARS-CoV-2 specific ribonucleic acid in samples for nucleic acid amplification detection**

**Short title: Degradation of SARS-CoV-2 specific RNA**

Katsuyuki Takeuchi^1,2^, Hiroyuki Yanagisawa^3^, Yukiko Kurosawa^3^, **Yoritsugu Iida^1^, Kosuke** **Kawai^3^**, and Shigehiko Fujimaki^3*^

^1^ Institute of Education, Innovative Human Resource Development Division, Tokyo Medical and Dental University, Bunkyo‐ku, Tokyo, Japan

^2^ Department of Genetics, Hyogo College of Medicine, Nishinomiya, Hyogo, Japan

^3^ Central Chemical Laboratory, SGS Japan Inc., Hodogaya-ku, Yokohama, Japan

*****Corresponding author

E-mail: shigehiko.fujimaki@sgs.com

Supporting information S1 Figure


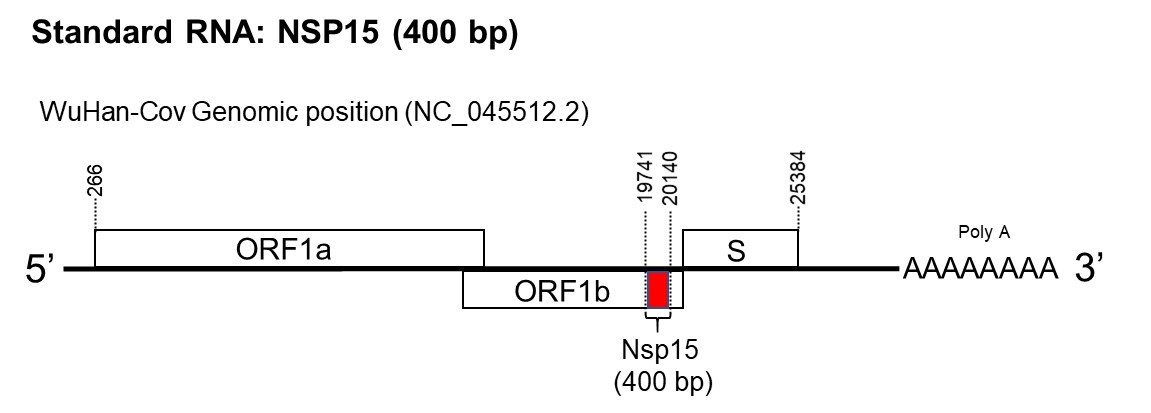

Supplement: S1 File — (DOCX) [file pone.0264541.s001.docx]
